# Seasonal trends in COVID-19 cases, hospitalizations, and mortality in the United States and Europe

**DOI:** 10.1038/s41598-023-31057-1

**Published:** 2023-03-08

**Authors:** Timothy L. Wiemken, Farid Khan, Laura Puzniak, Wan Yang, Jacob Simmering, Philip Polgreen, Jennifer L. Nguyen, Luis Jodar, John M. McLaughlin

**Affiliations:** 1 Pfizer Inc., 500 Arcola Rd., Collegeville, PA 19426 USA; 2 Columbia University Mailman School of Public Health-Epidemiology, 722 West 168th St., New York, NY 10032 USA; 3 Department of Internal Medicine-Pulmonary, Critical Care, and Occupational Medicine, University of Iowa, Iowa City, IA 52242 USA; 4 Department of Internal Medicine-Infectious Diseases, University of Iowa, Iowa City, IA 52242 USA

**Keywords:** Epidemiology, Epidemiology

## Abstract

Determining whether SARS-CoV-2 exhibits seasonality like other respiratory viruses is critical for public health planning. We evaluated whether COVID-19 rates follow a seasonal pattern using time series models. We used time series decomposition to extract the annual seasonal component of COVID-19 case, hospitalization, and mortality rates from March 2020 through December 2022 for the United States and Europe. Models were adjusted for a country-specific stringency index to account for confounding by various interventions. Despite year-round disease activity, we identified seasonal spikes in COVID-19 from approximately November through April for all outcomes and in all countries. Our results support employing annual preventative measures against SARS-CoV-2, such as administering seasonal booster vaccines in a similar timeframe as those in place for influenza. Whether certain high-risk individuals may need more than one COVID-19 vaccine booster dose each year will depend on factors like vaccine durability against severe illness and levels of year-round disease activity.

## Introduction

The Coronavirus Disease 2019 (COVID-19) pandemic has caused unprecedented worldwide morbidity, mortality, and social and economic disruption^1^. Globally, waves of infection, hospitalization, and mortality have paralleled the emergence of new variants of concern which have shown increased transmissibility^2^ and improved ability to evade vaccine- and infection-induced immunity^3^. Vaccination strategies to date have struggled to keep pace, and booster doses have been deployed to bolster protection against infection and symptomatic disease and maintain peak protection against severe disease throughout the pandemic^4–8^.

Many respiratory viruses show distinct seasonal patterns and result in waves of illness during the winter months^9,10^. These patterns are likely caused by a combination of host, pathogen, and environmental factors, including increased indoor activity and seasonal weather fluctuations known to impact viral stability outside the host^9,11–13^. Further, containment measures and pandemic virus variants have been shown to impact epidemic curves across various countries and could shift seasonal patterns^14,15^.

To date, there is still discussion about whether SARS-CoV-2 currently follows, or will follow in the future, similar seasonal patterns to other respiratory viruses^16–18^. Determining whether SARS-CoV-2 exhibits seasonal spikes in disease activity is critical for public health planning, including informing vaccination policy about the optimal timing for deploying additional doses. To help answer this urgent public health question, we used time series models to evaluate the seasonal patterns of COVID-19 cases, hospitalizations, and mortality in the United States and Europe with the objective to determine if COVID-19 follows typical respiratory virus seasonal patterns.

## Methods

### Primary analysis

We followed Strengthening the Reporting of Observational Studies in Epidemiology (STROBE) guidelines for our ecological study. Daily rates of COVID-19 cases, hospitalizations, and mortality per million population by country for the United States, all countries in the European Union, and the United Kingdom were obtained from the public-use Our World in Data (OWID) GitHub repository^19,20^. OWID sources case and mortality data from Johns Hopkins University^21^ and hospitalization data from various official country-specific sources^18^. Data were included from 01 Mar 2020 through 31 Dec 2022, as available by country. If daily data were missing or unavailable, data were substituted from that country’s corresponding weekly data.

### Statistical analysis

We used a Prophet time series model to decompose daily country-specific time series rates of COVID-19 cases, hospitalizations, and mortality separately and adjusted for the country stringency index in an additive, linear time series^22,23^. In these models, we decomposed the observed data into a trend component and weekly and annual seasonal components and included the country-specific stringency index as a regressor to account for potential confounding by nonpharmaceutical interventions. The stringency index was developed by the Oxford Coronavirus Government Response Tracker project and is a composite of nine metrics including school closures, workplace closures, cancellation of public events, restrictions on public gatherings, closures of public transport, stay-at-home requirements, public information campaigns, restrictions on mobility and access to services, and international travel restrictions^24^. Uncertainty interval widths for computed point estimates were set to 95%, with 2000 uncertainty samples used to compute the intervals. Bayesian sampling was performed with 2000 Markov Chain Monte Carlo samples to obtain uncertainty intervals for seasonality estimates. In our models, we focused our analysis on the annual seasonal component only, which models the annual seasonal changes in observed rates after any long-term trends. The estimated rate is the sum of the long-term trend, weekly, and seasonal components, as well as the estimated effect of the country-specific stringency index. Positive values for the seasonal component indicate rates during that time of year are higher than during other parts of the year, while negative values indicate decreased rates during that season.

In addition to extracting the decomposed trend, annual seasonal component, weekly seasonal component, and the estimated effect of the stringency index, the monthly median value of the annual seasonal component was computed and displayed in a country-level heatmap to depict the months driving annual seasonal increases more clearly.

### Modeling validation

To validate our modeling approach, we applied the same Prophet model (excluding the stringency index regressor) to pre-pandemic country-level influenza data from the World Health Organization^25^. This approach was used to assess whether the model would predict well-documented annual seasonal patterns for influenza and if those patterns matched those we documented for SARS-CoV-2. Influenza positivity rates were defined by computing the percent positive specimens of all respiratory specimens processed from 04 Oct 2009 through 19 Dec 2021 using the same countries (the United States, European Union countries, and the United Kingdom). Malta was not evaluated, as influenza data were not available from the World Health Organization.

### Human subjects protection

All data used in this study were publicly available and de-identified and therefore did not constitute human subjects research per 45 CFR 46.102. Due to this, the study did not require IRB registration or review.

R version 4.2.2 (R Foundation for Statistical Computing, Vienna, Austria) was used for all analyses.

## Results

**Figure 1:**
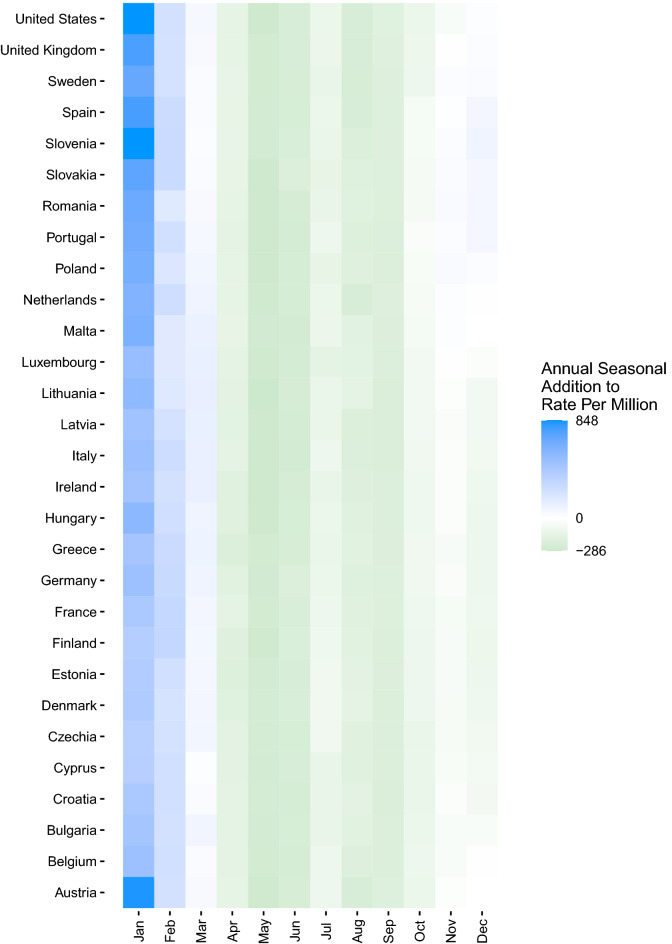

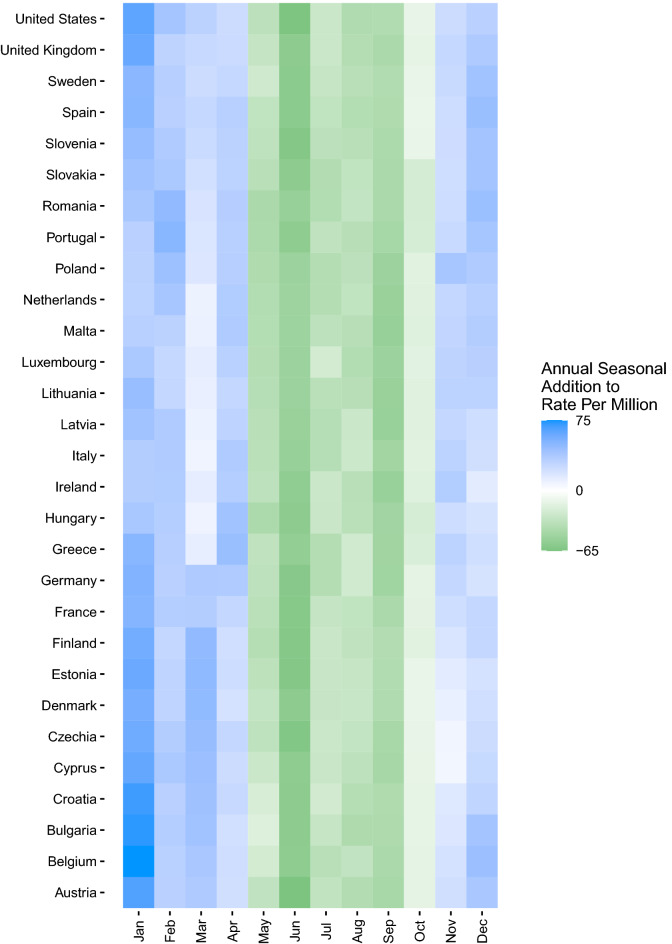

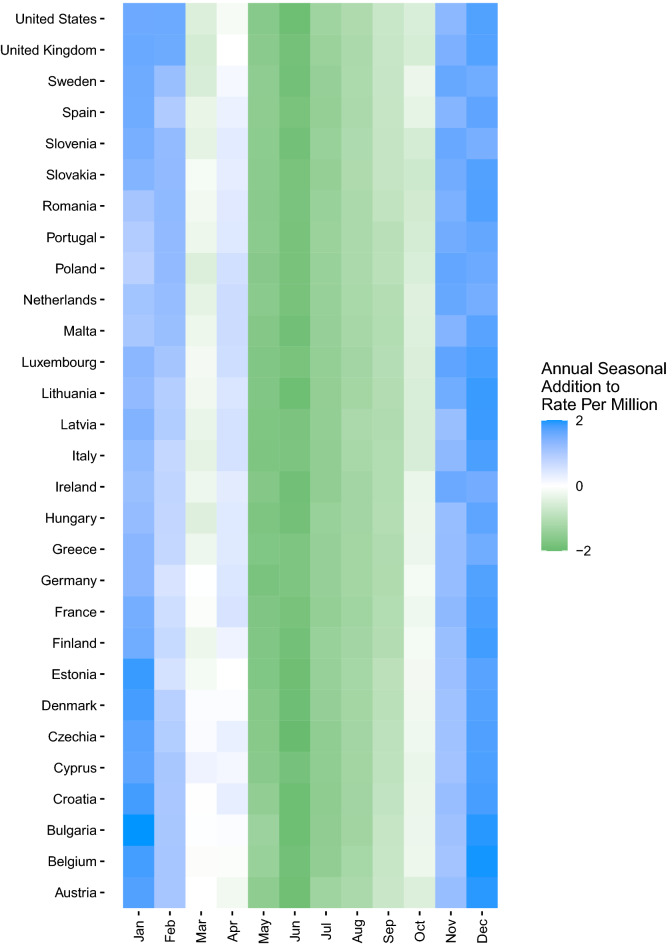
Heatmap of COVID-19 median annual seasonal components by month by country, 01 Mar 2020–31 Dec 2022. (**A**) Cases per million population; (**B**) COVID-19 hospitalizations per million population. (**C**) COVID-19 mortality per million population. Data Source: Our World in Data^19,20^.

Collapsed across all years of our study period, our model showed distinct annual seasonality consistent across COVID-19 case (Fig. 1a), hospitalization (Fig. 1b), and mortality (Fig. 1c) rates from approximately November through April. The additional impact of the annual seasonal component for cases was most substantial in January through March, where there was an additional 848 COVID-19 cases per 1,000,000 population. The annual seasonal component for hospitalizations indicated a clear differentiation between seasons, with up to 75 additional hospitalizations per 1,000,000 from November through April. COVID-19-associated mortality showed a similar trend in the annual seasonal component, with an additional two deaths per 1,000,000 population due to the annual seasonal effect primarily occurring from November through February. These trends were consistent in all countries evaluated.

Total rates of COVID-19 cases, hospitalizations, and mortality, as well as influenza positivity, collapsed across all countries by month and year can be seen in Supplementary Figs. s1–s4. Country-specific time-series annual seasonal components not aggregated by month, along with other individual decomposed components (trend, weekly seasonality, and stringency index), can be seen in Supplementary Figs. s5–s8 (cases), Supplementary Figs. s9–s12 (hospitalizations), and Supplementary Figs. s13–s16 (mortality). Most of the positive annual seasonality values were within the typical respiratory viral season in the Northern Hemisphere and are indicated with a purple highlight in the annual seasonal component Supplementary Figs. s5, s9, and s13.

**Figure 2:**
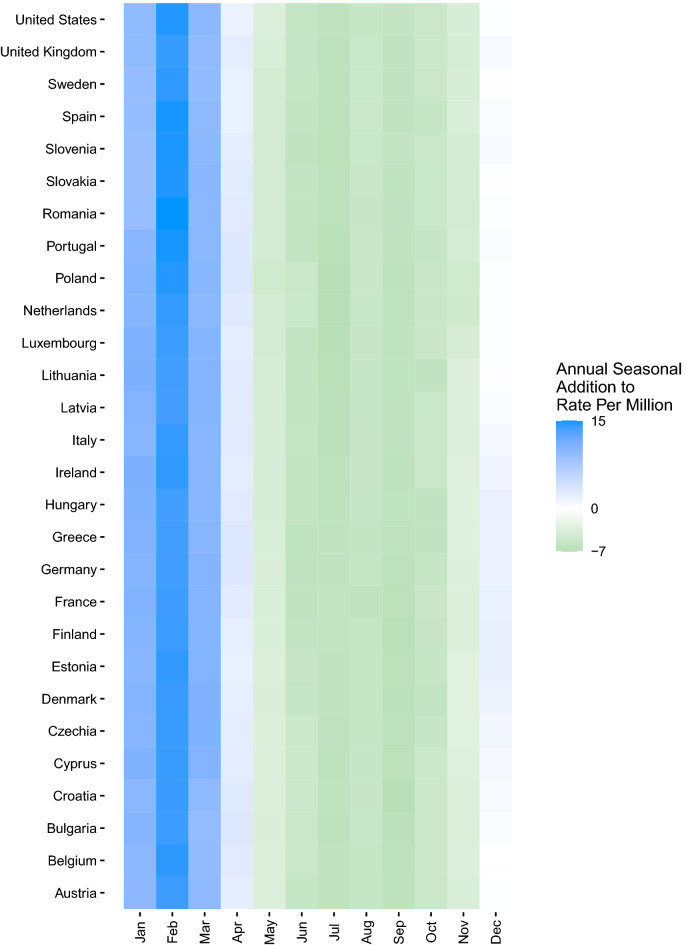
Heatmap of the influenza percent positivity median annual seasonal component by month, Oct 2009 through Dec 2021. Data Source: World Health Organization^25^.

Our analyses using the exact model specifications described for rates of COVID-19 (excluding the stringency index regressor) also documented the well-established annual seasonality of pre-pandemic influenza between December and April over twelve US influenza seasons (Fig. 2), consistent with current knowledge regarding annual influenza patterns in the Northern Hemisphere temperate regions^26^.

## Discussion

Although SARS-CoV-2 continues to cause disease throughout the year, we identified seasonal spikes in COVID-19 cases, hospitalizations, and mortality from November through April across all years of the pandemic to date in the United States and Europe, a finding that is consistent with the typical months of seasonal respiratory virus epidemics in the northern hemisphere^27^. Our results indicate seasonal spikes are consistent with seasonal patterns seen for influenza^27^, respiratory syncytial virus (RSV)^28^, and other coronaviruses^29^, and are compatible with mathematical simulations of COVID-19 activity^16,17^.

There are many possible reasons for the seasonality of respiratory viruses, including climate-related changes in viral transmissibility, modified host factors (e.g., waning of infection- or vaccine-induced immunity), and changes in human behavior during the winter months^9,18^. Regardless of the mechanisms, knowledge of pathogen seasonality is imperative for instituting targeted interventions to lessen the impact when the burden on our healthcare infrastructure is the greatest. Accordingly, our findings have important vaccine policy implications. Additional doses of COVID-19 vaccines or modified versions of the vaccines administered before the winter months will likely have the most significant public health impact on the COVID-19 burden. This is analogous to providing influenza vaccine before peak flu activity each year to mitigate the largest spikes in disease burden. Despite evidence that protection provided by current mRNA COVID-19 vaccines wanes significantly against omicron infection and symptomatic disease after only 3–4 months, even after a booster^5,8,30^, this short-term added protection could still provide meaningful defense against SARS-CoV-2 infection if deployed just before seasonal waves which last 3–4 months on average.

Because SARS-CoV-2 appears to be more transmissible than influenza and other seasonal respiratory viruses, it seems likely that year-round SARS-CoV-2 activity will remain elevated compared to other pathogens^31^. COVID-19 continues to cause substantial morbidity and mortality throughout the year, including outside of the traditional viral respiratory season. In addition, rapid evolution of new variants or subvariants could impact seasonal patterns. Our data showed smaller waves of COVID-19 in the summer months, which were likely driven by new variants that emerged during this time period over the course of the pandemic (i.e., the delta variant in summer 2021 and the omicron subvariant BA.4/5 in summer 2022)^32^. Novel variants or subvariants that exhibit enhanced immune escape or transmissibility or any other property that increases viral fitness could alter seasonal patterns or cause an off-season outbreak^33^. Thus, additional COVID-19 vaccine booster doses may be needed at a frequency greater than once annually for certain high-risk individuals. This determination will be a careful balance between epidemiological, benefit-risk, and programmatic considerations (including concerns regarding “booster fatigue”^34,35^) moving forward and will likely depend primarily on COVID-19 vaccine durability against severe illness and levels of year-round disease activity. For this reason, continued surveillance of real-time vaccine performance and the emergence of new variants remains critical.

It should be noted that other viral pathogens also have, on occasion, followed atypical seasonal patterns. For example, the 2009 H1N1 influenza pandemic began in the spring of 2009 toward the end of the typical influenza season in the Northern Hemisphere^36^. A pandemic was declared on June 11, 2009 and cases peaked in July. It was not until the second autumn wave that disease patterns became more aligned with the typical influenza season^36^. In addition, pandemic containment measures can impact seasonal trends. For example, during the COVID-19 pandemic, both influenza and respiratory syncytial virus transmission followed atypical patterns^37^. Further, at the time of writing, China is currently experiencing a large wave of COVID-19 that likely corresponds with the lifting of country-wide lockdown measures^38^ Vaccination could also shift the seasonality of respiratory viruses, however, this has not occurred for influenza, the only other respiratory virus for which vaccination is available and uptake is high.

Our methodology also detected the annual seasonality of influenza virus in the same countries, corresponding to known annual seasonal patterns of influenza^27,28^, underscoring the utility of the methodology we used for detecting seasonal patterns in common respiratory viruses. Regardless, our results have at least five limitations. First, we could not account for the potential underreporting of cases, which may have a large effect more recently with increases in at-home SARS-CoV-2 testing that may not be reported^39^. Finding similar results for COVID-19 hospitalizations and deaths, which are less likely to be under-reported, however, was reassuring. Second, statistical modeling may not fully reflect the intricacies of preventing transmissible infectious diseases, such as the impact of COVID-19 vaccination, waning immunity, or changes in testing, nonpharmaceutical interventions, or healthcare-seeking behavior over time. Third, although the pandemic is in its third year, the longitudinal data available for modeling was limited compared to other common seasonal viruses. Because of this, similar models created in the future may illustrate different outputs given variable prevention behaviors, vaccines and vaccine uptake, and novel SARS-CoV-2 variants. Fourth, our findings are not generalizable beyond the United States and Europe. More research is needed to understand if the same annual seasonal patterns in SARS-CoV-2 activity are seen in the Southern Hemisphere or Asia–Pacific regions. Finally, with SARS-CoV-2, there is always the potential for new variants to emerge that could meaningfully escape prior vaccine- or infection-induced immunity and cause significant epidemics outside of regular seasonal patterns identified thus far in the pandemic. Therefore, the public health community should continue to plan and maintain the capability for sufficient response in the event of this possibility.

## Conclusion

Our study suggests that COVID-19 activity and associated hospitalization and mortality in the United States and Europe peak during the traditional winter viral respiratory season despite continual transmission throughout the year. Thus, employing annual protective measures against SARS-CoV-2 such as administering seasonal booster vaccines or other non-pharmaceutical interventions for the general population in a similar timeframe as those already in place for influenza prevention (i.e., beginning in early autumn) is a prudent strategy to stay ahead of likely forthcoming seasonal waves of COVID-19. However, whether certain high-risk individuals may need more than one booster dose each year will depend on factors like vaccine durability against severe illness and levels of year-round disease activity. Additional confirmatory studies are needed, including those conducted in the Southern Hemisphere and other regions outside the United States and Europe.

## Supplementary Information

Supplementary Figures.

## Data Availability

All data utilized in this study are open and available to the public, as referenced in the manuscript. Reasonable requests for programming scripts can be made to the corresponding author.
