# Supplementary material for: Seasonal trends in COVID-19 cases, hospitalizations, and mortality in the United States and Europe
Source: Sci Rep. 2023 Mar 8;13:3886. doi: 10.1038/s41598-023-31057-1 (PMC9994397; doi:10.1038/s41598-023-31057-1)
Supplement: Supplementary file 1 — Supplementary Figures. [file 41598_2023_31057_MOESM1_ESM.pdf]

Supplementary Appendix: Seasonal trends in COVID-19 cases, hospitalizations, and deaths in the United States and Europe

Figure 1: COVID-19 case rate per million population by month-year, February 2020 – December 2022, European Union, United Kingdom, and United States 3

Figure 2: COVID-19 hospitalization rate per million population by month-year, February 2020 – December 2022, European Union, United Kingdom, and United States 4

Figure 3: COVID-19 mortality rate per million population by month-year, February 2020 – December 2022, European Union, United Kingdom, and United States 5

Figure 4: Influenza percent positivity October 2009 - December 2021, European Union, United Kingdom, and United States 6

Figure 5: Annual seasonal component for COVID-19 cases, March 2020 - December 2022....... 7

Figure 6: Trend component for COVID-19 cases, March 2020 - December 2022 8

Figure 7: Weekly seasonal component for COVID-19 cases, March 2020 - December 2022 9

Figure 8: Stringency Index component for COVID-19 cases, March 2020 - December 2022.... 10

Figure 9: Annual seasonal component for COVID-19 hospitalizations, March 2020 - December 2022 11

Figure 10: Trend component for COVID-19 hospitalizations, March 2020 - December 2022... 12

Figure 11: Weekly seasonal component for COVID-19 hospitalizations, March 2020 - December 2022 13

Figure 12: Stringency Index component for COVID-19 hospitalizations, March 2020 - December 2022 14

Figure 13: Annual seasonal component for COVID-19 mortality, March 2020 - December 2022 15

Figure 14: Trend component for COVID-19 mortality, March 2020 - December 2022 16

Figure 15: Weekly seasonal component for COVID-19 mortality, March 2020 - December 2022 17

Figure 16: Stringency Index component for COVID-19 mortality, March 2020 - December 2022 18

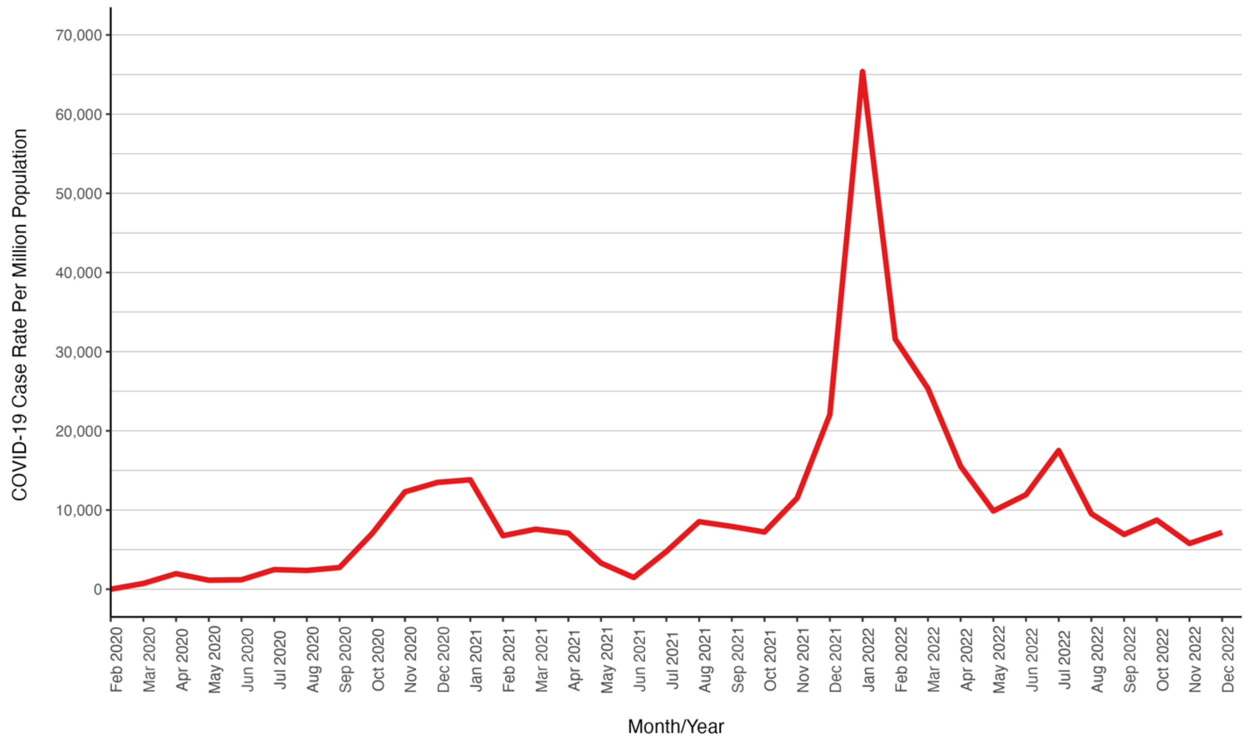

Figure 1: COVID-19 case rate per million population by month-year, February 2020 – December 2022, European Union, United Kingdom, and United States

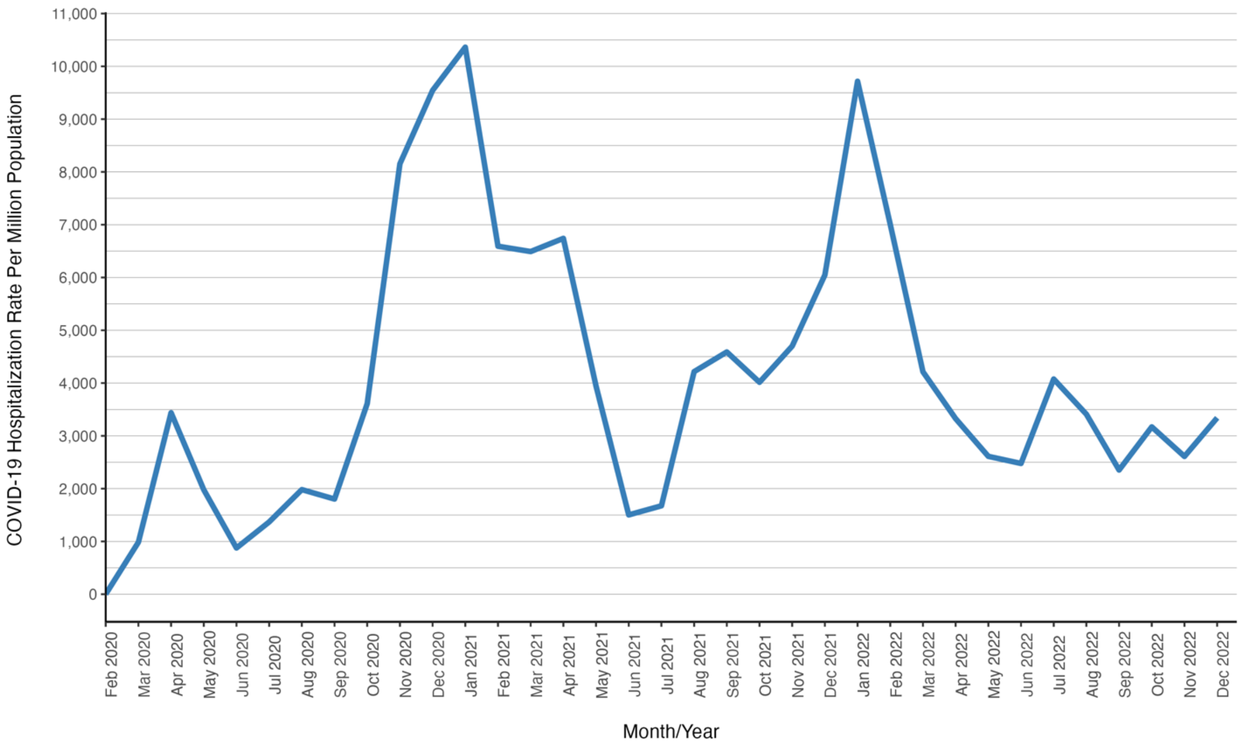

Figure 2: COVID-19 hospitalization rate per million population by month-year, February 2020 – December 2022, European Union, United Kingdom, and United States

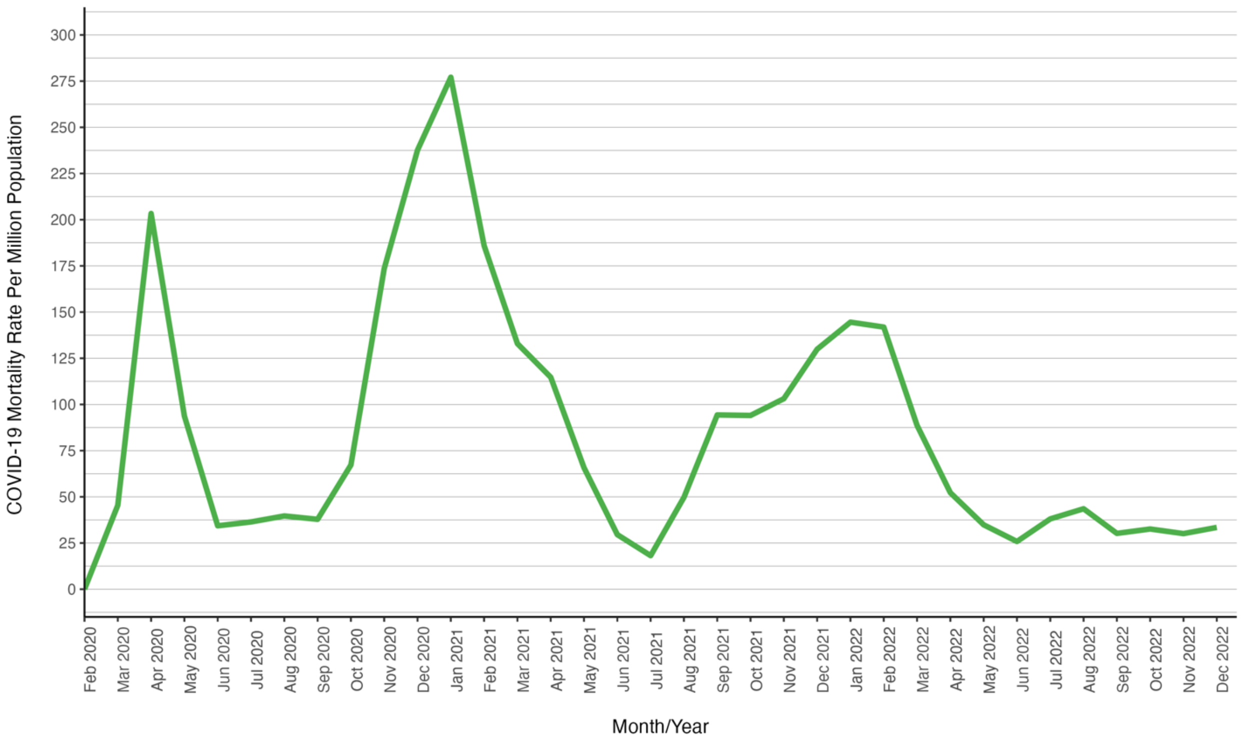

Figure 3: COVID-19 mortality rate per million population by month-year, February 2020 – December 2022, European Union, United Kingdom, and United States

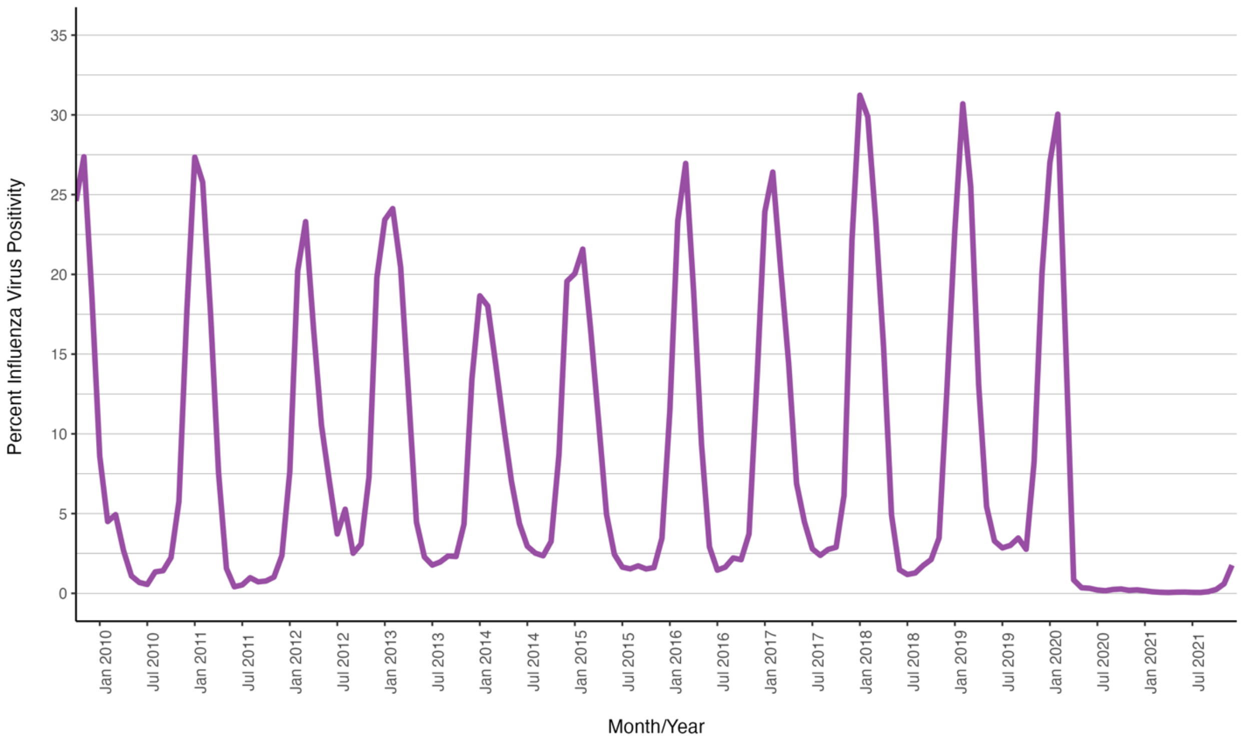

Figure 4: Influenza percent positivity October 2009 - December 2021, European Union, United Kingdom, and United States

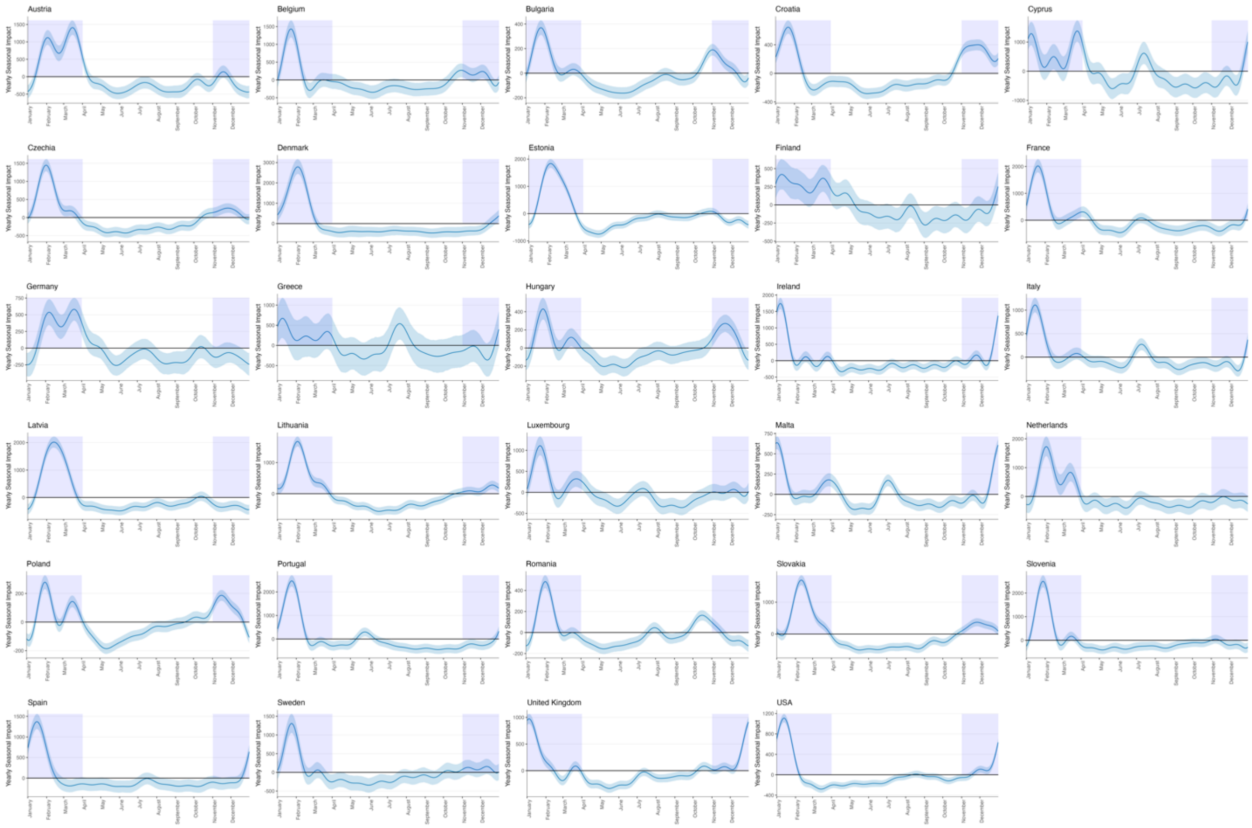

Figure 5: Annual seasonal component for COVID-19 cases, March 2020 - December 2022

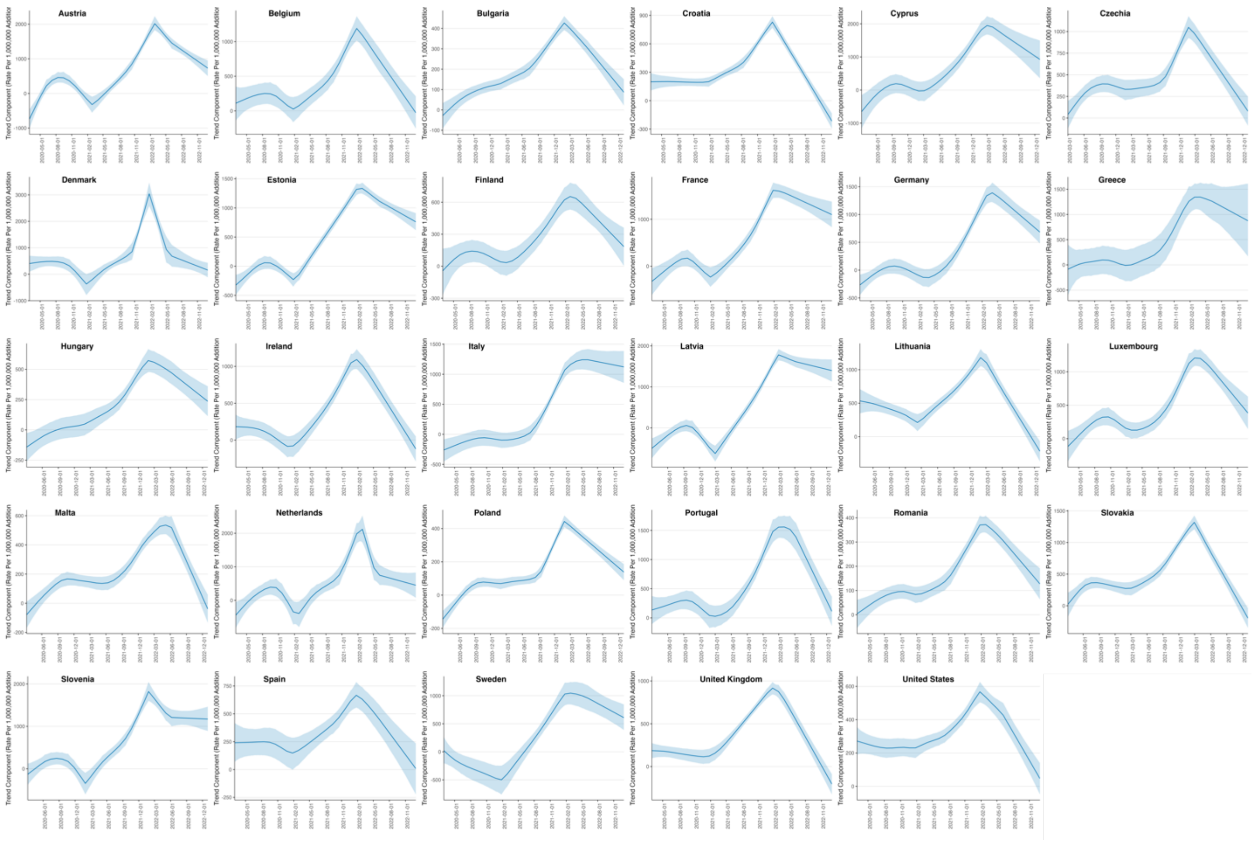

Figure 6: Trend component for COVID-19 cases, March 2020 - December 2022

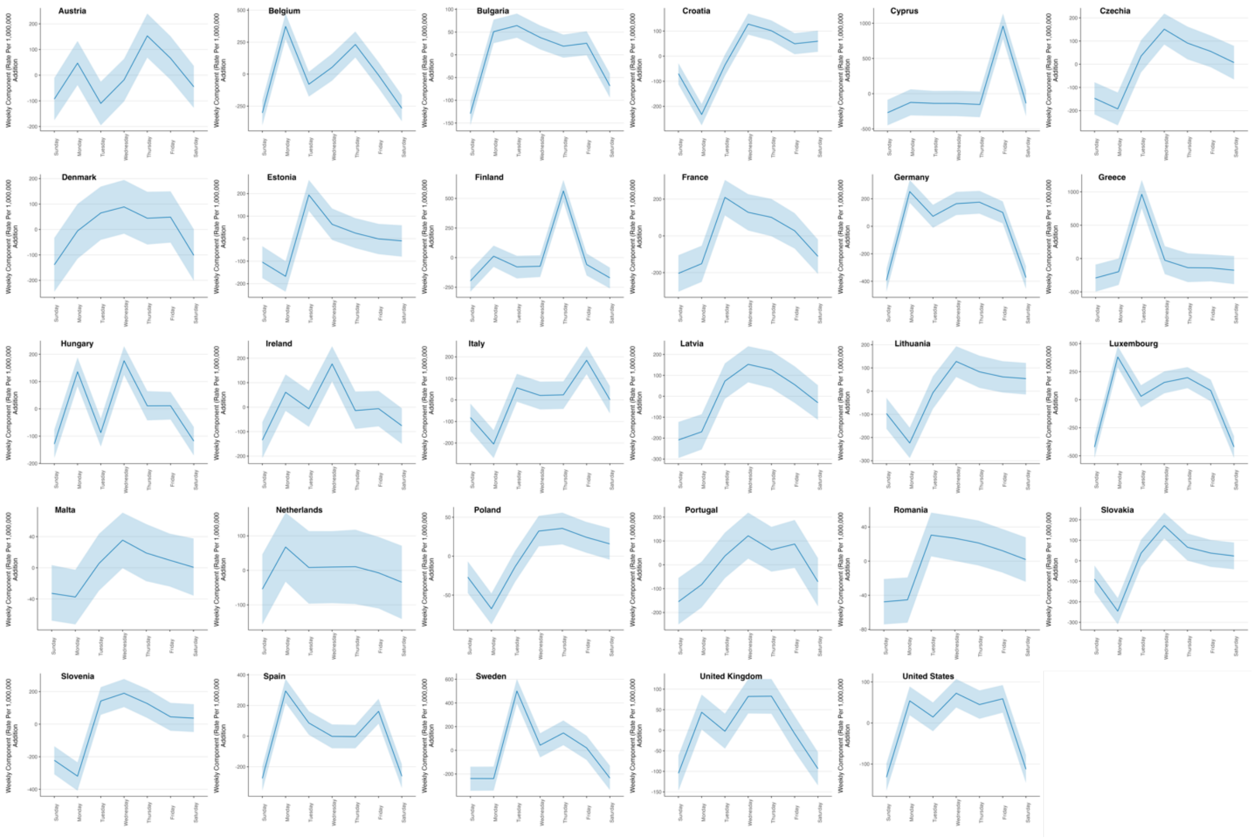

Figure 7: Weekly seasonal component for COVID-19 cases, March 2020 - December 2022

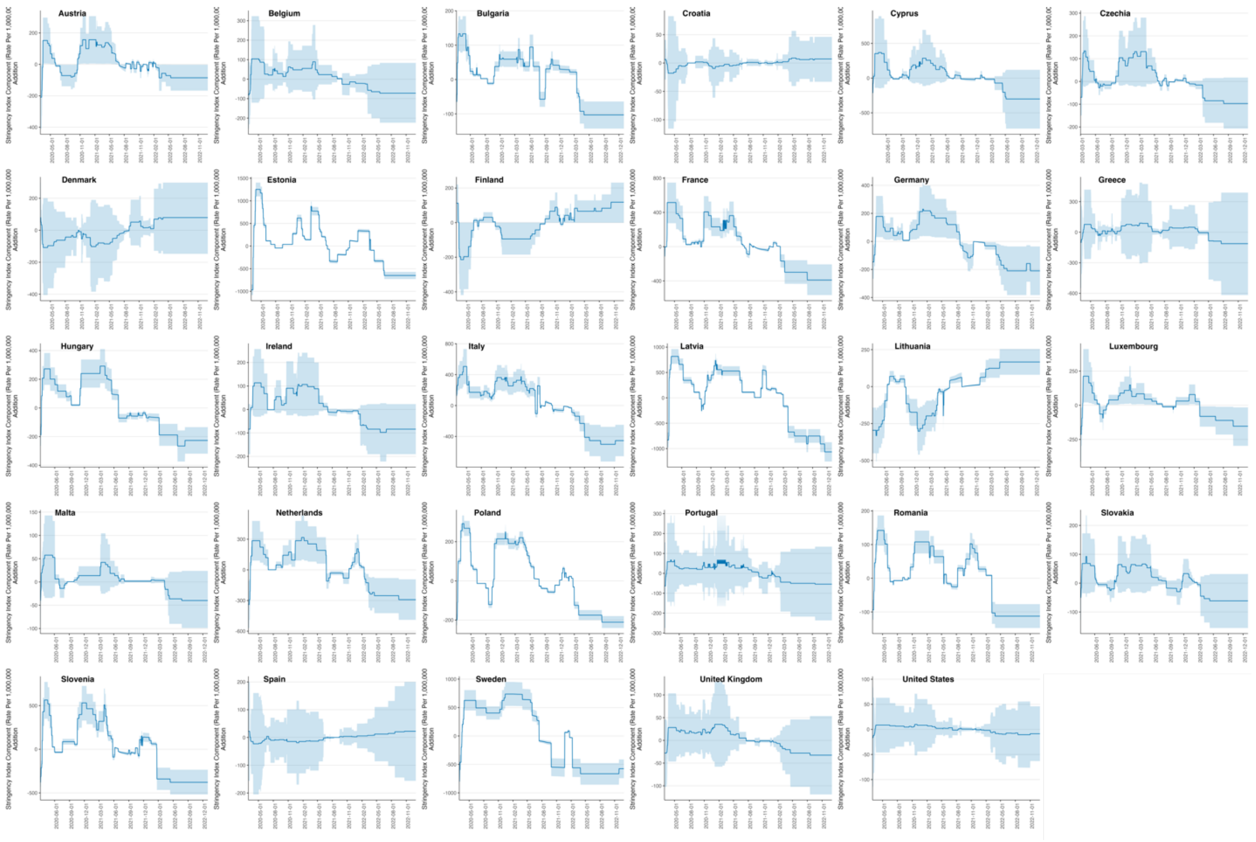

Figure 8: Stringency Index component for COVID-19 cases, March 2020 - December 2022

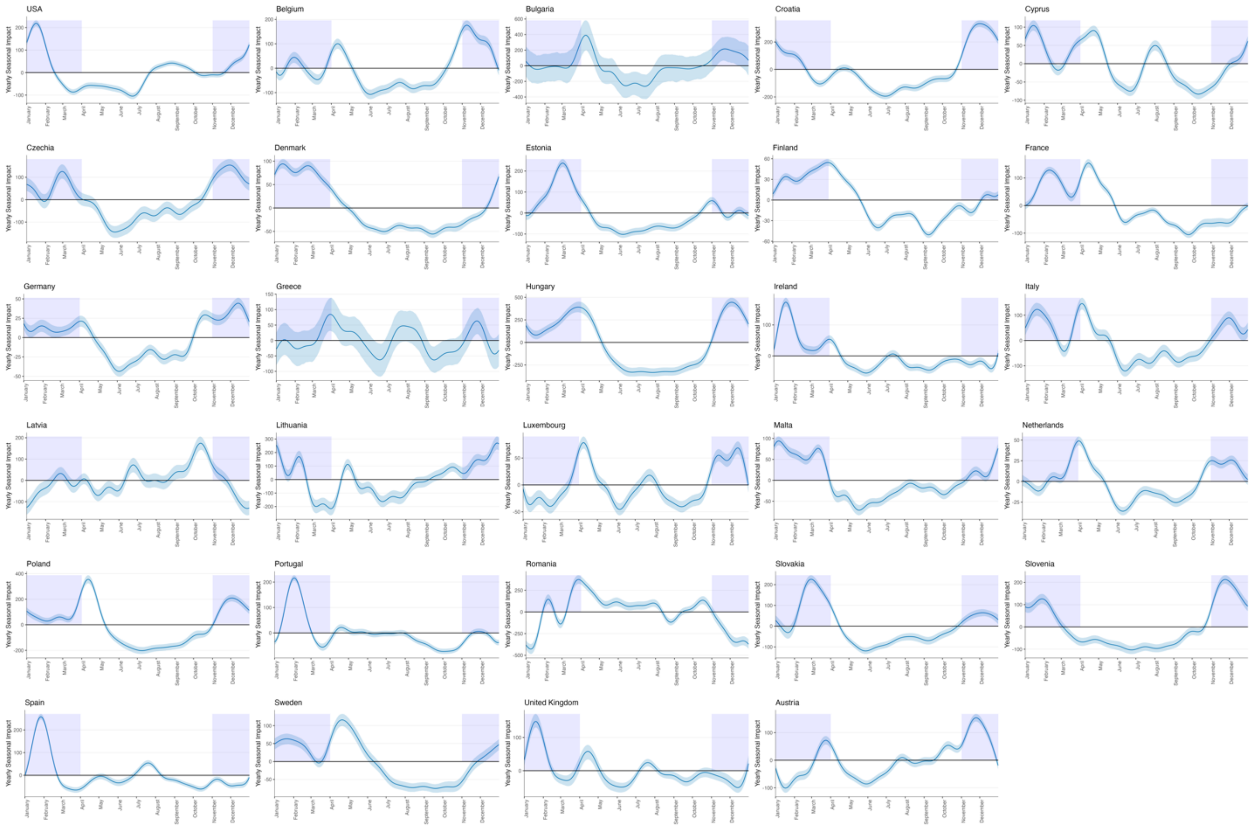

Figure 9: Annual seasonal component for COVID-19 hospitalizations, March 2020 - December 2022

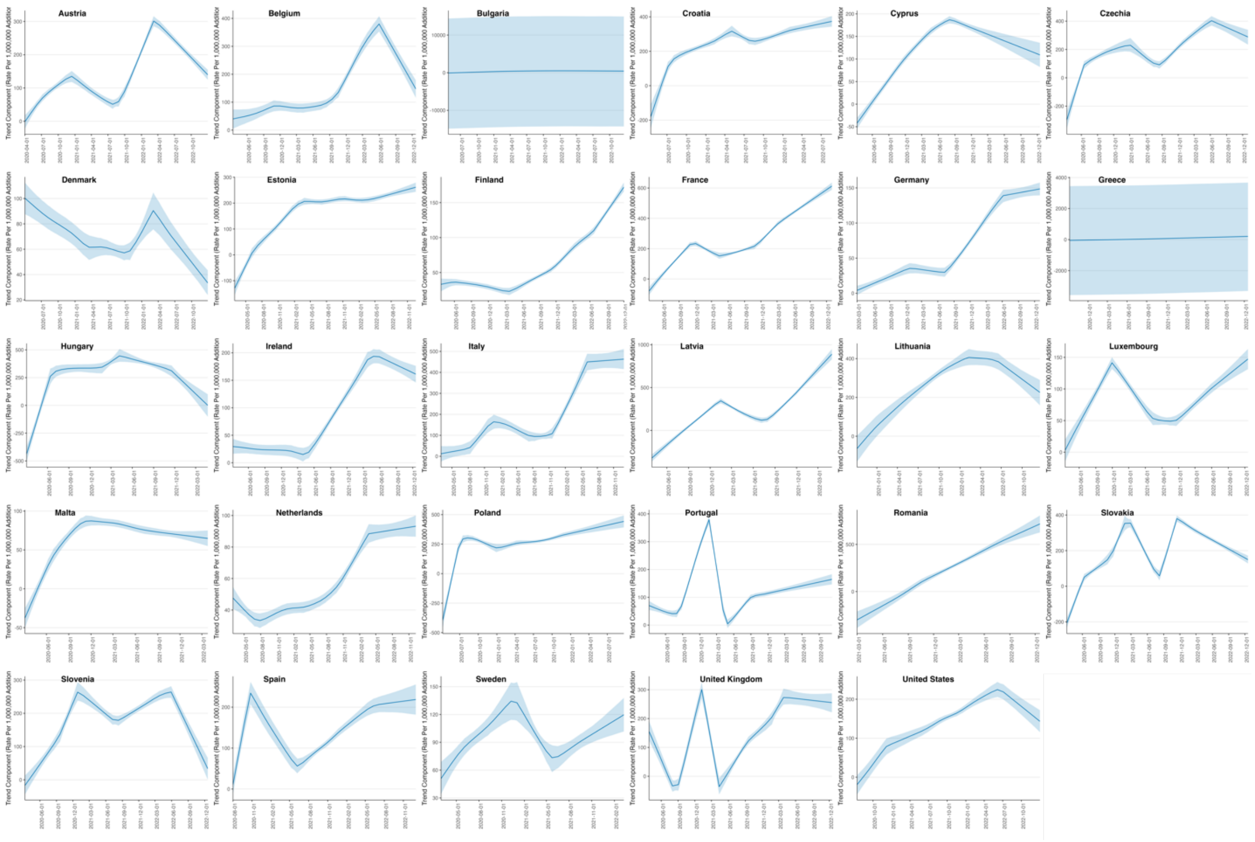

Figure 10: Trend component for COVID-19 hospitalizations, March 2020 - December 2022

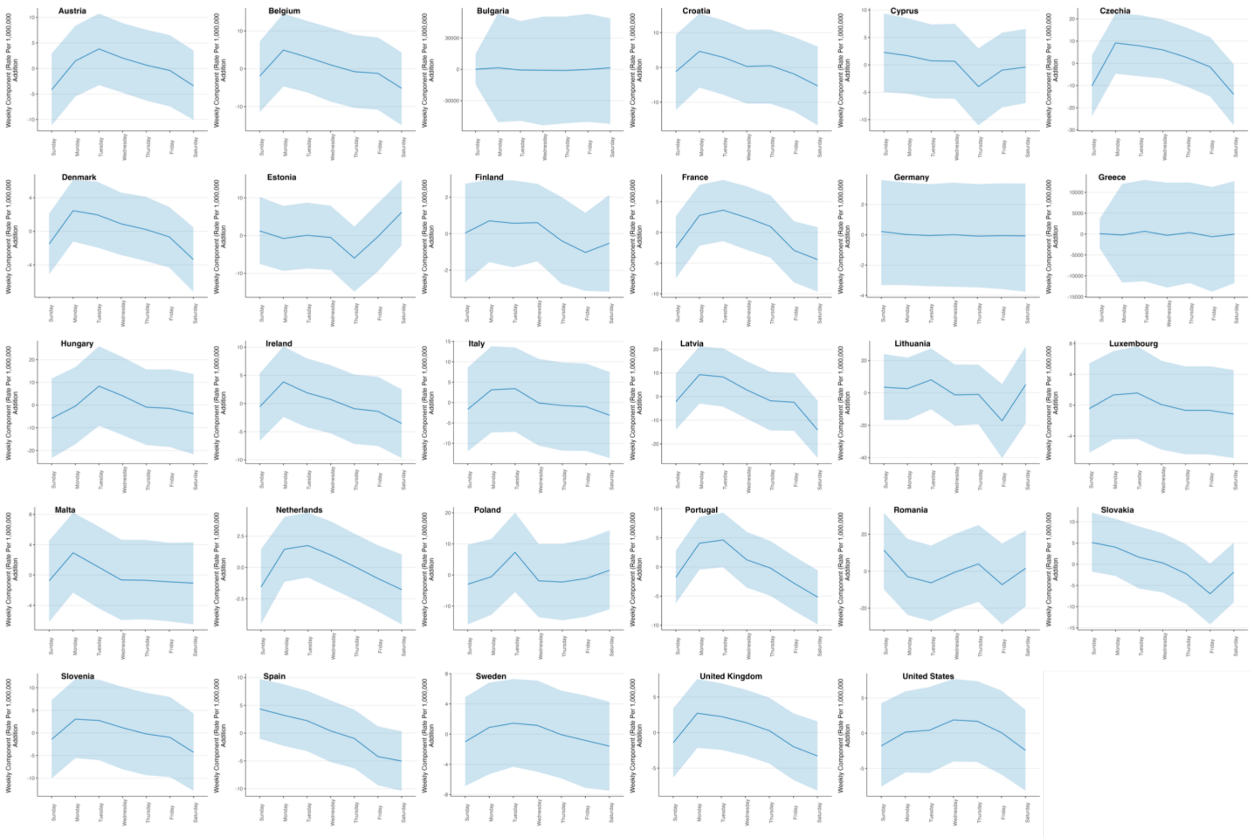

Figure 11: Weekly seasonal component for COVID-19 hospitalizations, March 2020 - December 2022

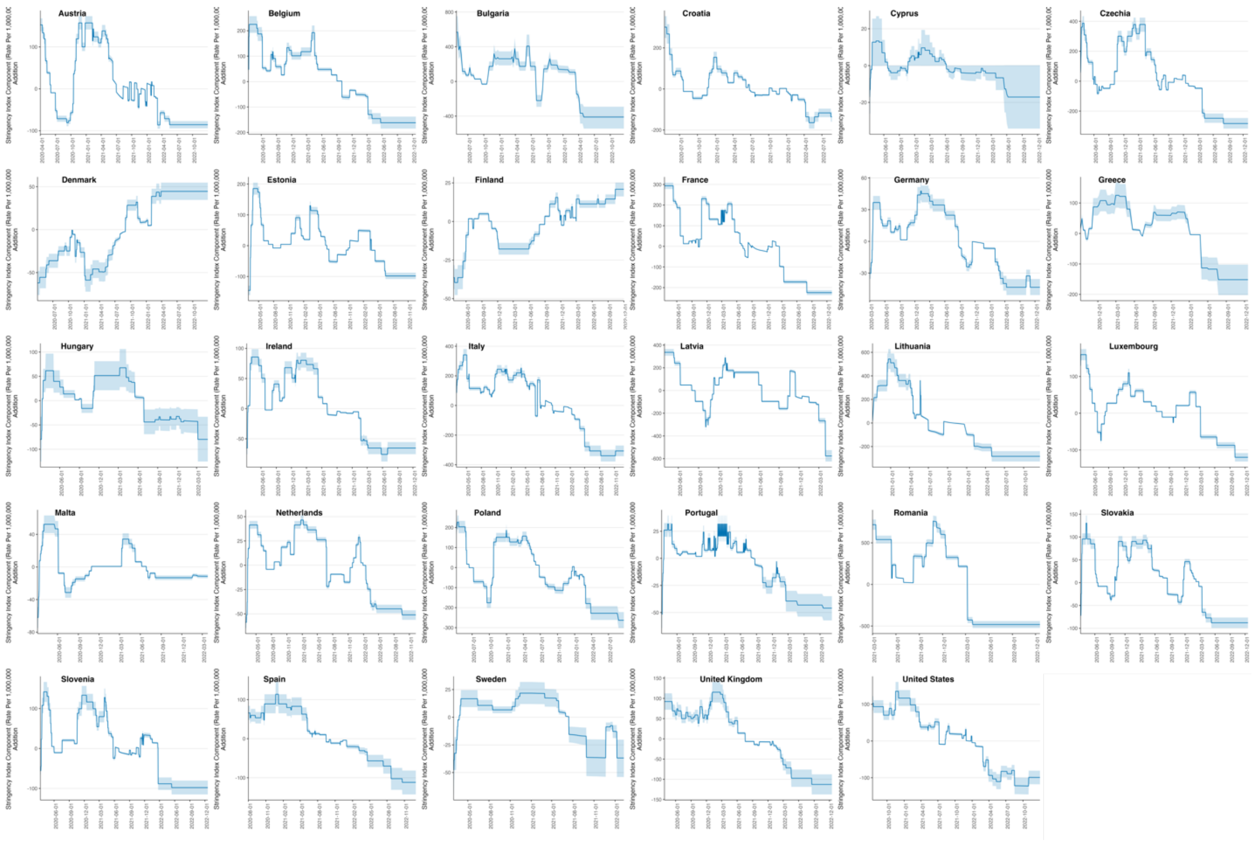

Figure 12: Stringency Index component for COVID-19 hospitalizations, March 2020 - December 2022

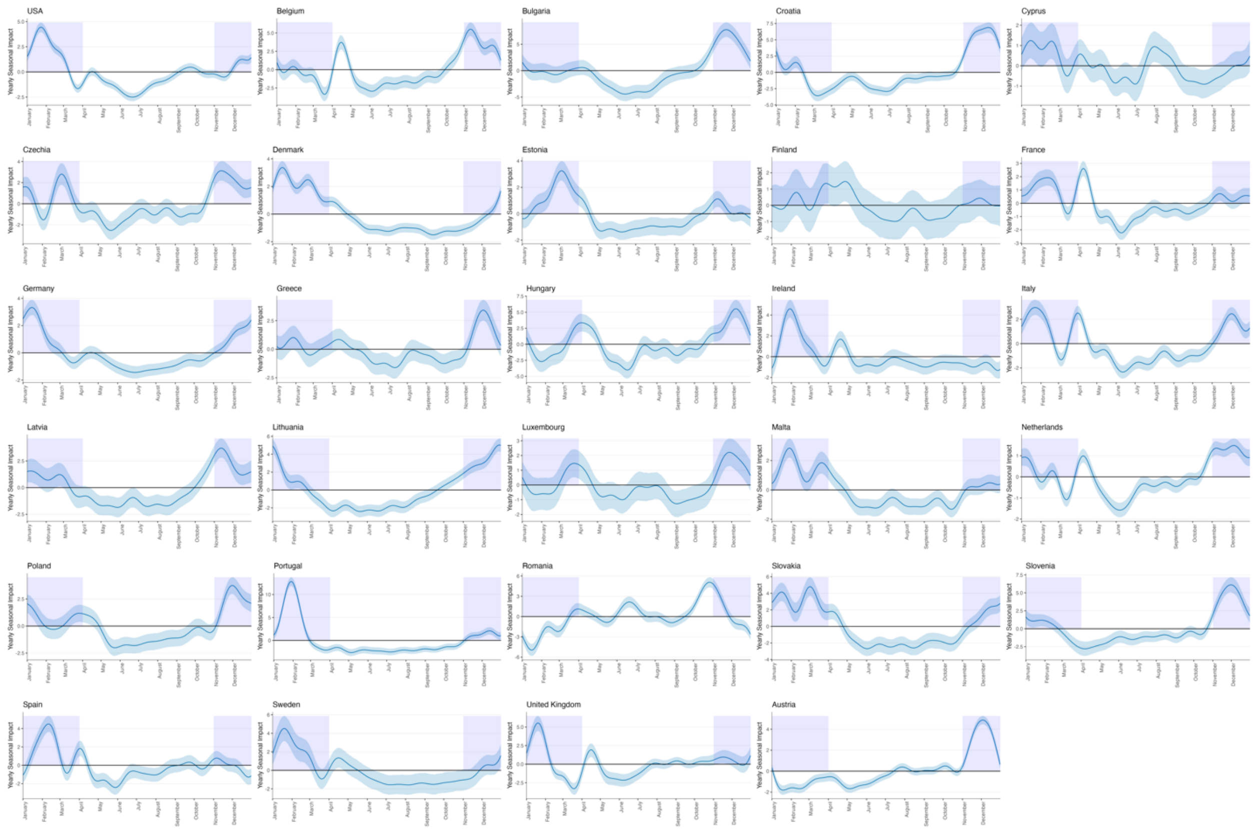

Figure 13: Annual seasonal component for COVID-19 mortality, March 2020 - December 2022

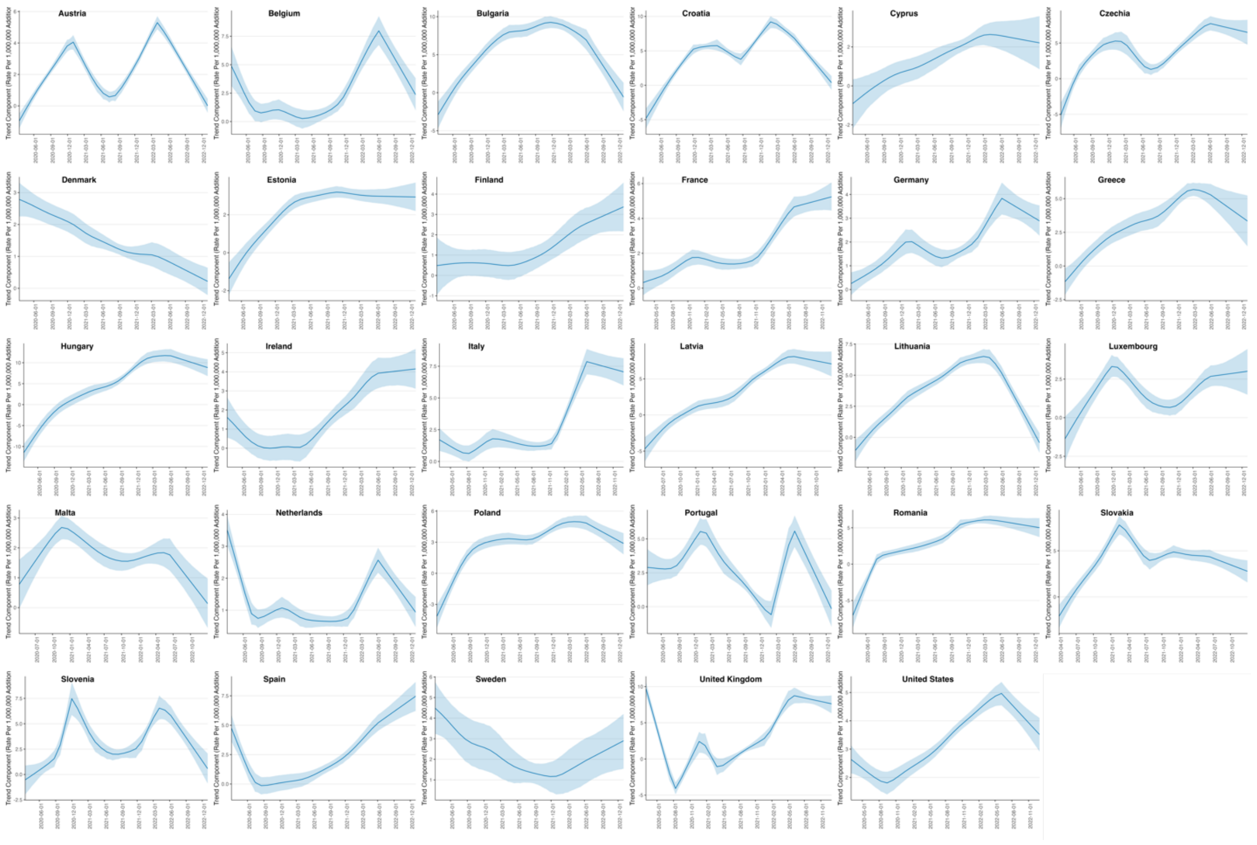

Figure 14: Trend component for COVID-19 mortality, March 2020 - December 2022

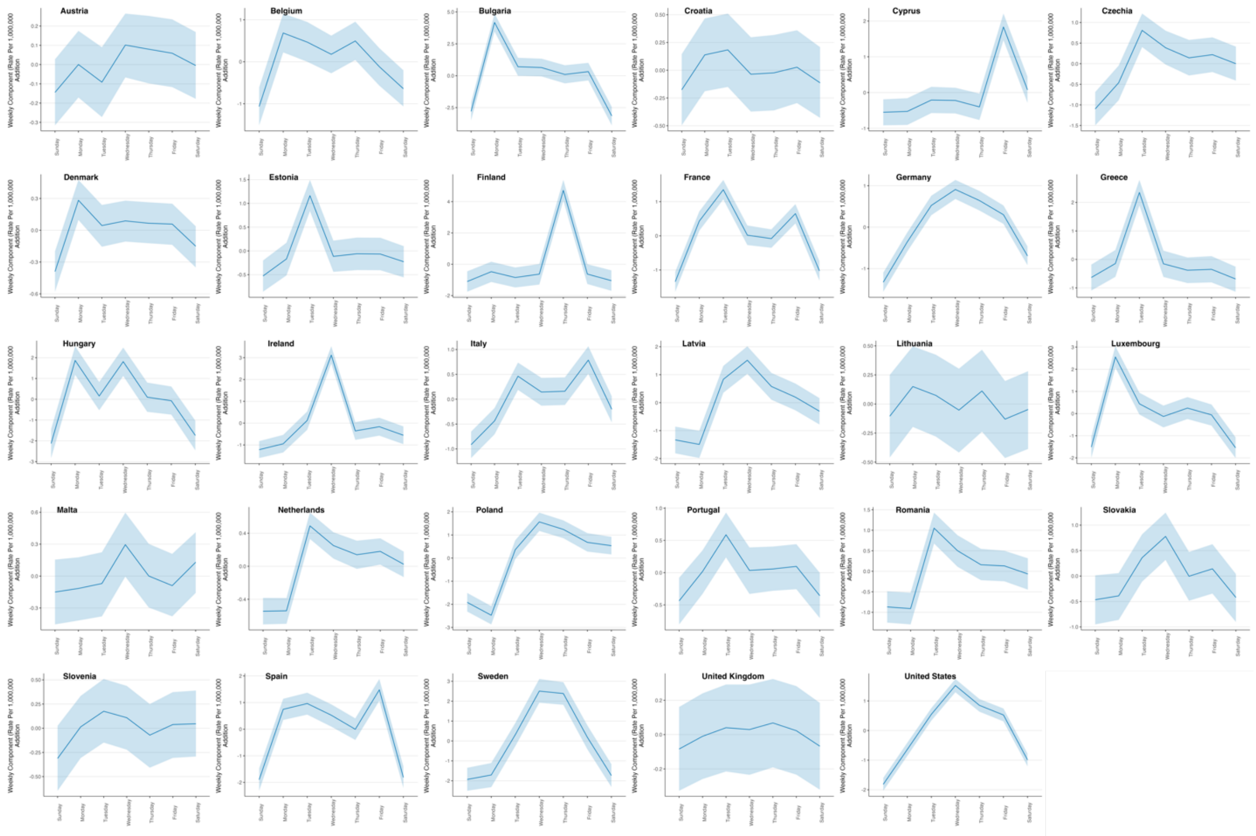

Figure 15: Weekly seasonal component for COVID-19 mortality, March 2020 - December 2022

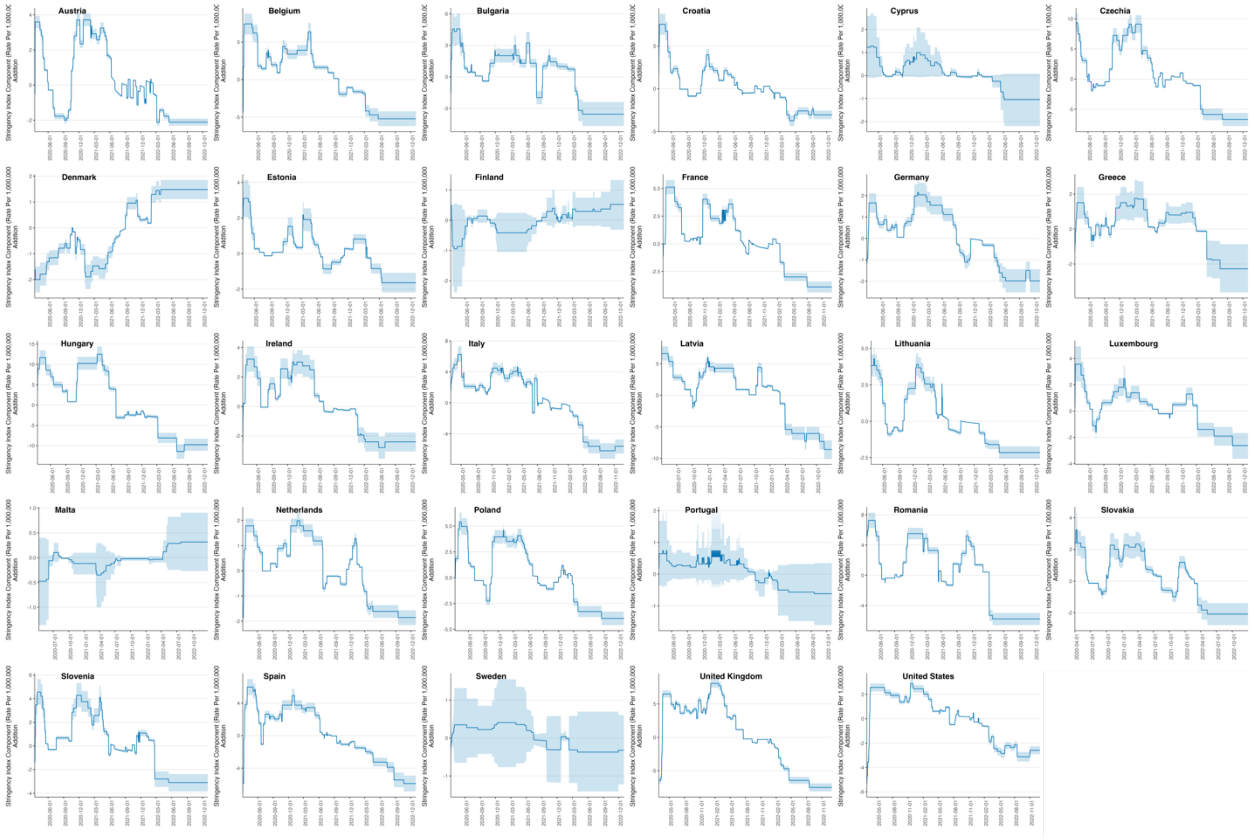

Figure 16: Stringency Index component for COVID-19 mortality, March 2020 - December 2022
